# Keeping Patients on Their Feet: How Fall Prevention Clinics Can Be More Effective

**DOI:** 10.1007/s11606-026-10276-y

**Published:** 2026-02-27

**Authors:** David R. Lee, Lauren K. Matsuno, Joyce H. Seo, Betty Y. Wong, Erin A. Cook, Grace I. Chen, Kyle Newbrey, Susan Stich, David B. Reuben

**Affiliations:** 1 Department of Medicine, David Geffen School of Medicine, University of California, Los Angeles, CA USA; 2 Brandman Centers for Senior Care, Los Angeles, CA USA; 3 UCLA Health, Outpatient Rehabilitation, Los Angeles, CA USA

**Keywords:** fall prevention, quality improvement, physical therapy, bone health, interdisciplinary team

## Abstract

**Background:**

Although fall prevention programs in real-world clinical settings often generate recommendations to reduce fall risk factors, evidence that they improve clinical outcomes remains limited. To better understand this gap, we examined implementation rates of recommendations.

**Methods:**

This retrospective cohort study evaluated patients who received care at an interdisciplinary (nurse, physical therapist, geriatrician) fall prevention consultation clinic from November 2020 to December 2022. Data were collected on patient demographics, screening assessments (including self-reported falls, fracture risk, visual acuity, cognition, and orthostatic blood pressure), physical therapy evaluations, and implementation of recommendations. Fall- and fracture-related ED visits and hospitalizations in the year before and after the clinic visit were compared.

**Results:**

Ninety-four patients seen with a mean age of 79 years (SD = 8.4, range 63–101); 71% were women, 60% self-identified as non-Hispanic White, 39% needed help with ≥ 1 activity of daily living, and 62% needed help with ≥ 2 instrumental activities of daily living. Patients screened positive for a self-reported fall in the last year (81%), increased fracture risk (67%), vision impairment (43%), cognitive concerns (27%), and orthostatic hypotension (12%). Implementation of recommendations occurred most often for home (58%) and outpatient (46%) physical therapy, bisphosphonate recommendations or orders (50%), ophthalmology (43%), and medication adjustment recommendations (36%). Referrals to podiatry (30%), clinical pharmacy (29%) for fall risk-increasing drugs, osteoporosis clinic (23%), and memory clinic (12%) were implemented least frequently. There were no statistically significant differences in fall- or fracture-related ED visits (IRR 0.61, 95% CI 0.33–1.15) or hospitalizations (IRR 0.75, 95% CI 0.32–1.72) 1-year pre- and post-clinic evaluation.

**Conclusions:**

Although this consultative fall prevention clinic generated actionable recommendations, their implementation was sub-optimal. The study identified key areas for process improvement, particularly enhancing adherence to high-impact recommendations such as physical therapy and bone health.

## INTRODUCTION

Falls are leading causes of injury, hospitalization, and mortality among adults aged 65 and older.^1,2^ Approximately one in three older adults in the community report at least one fall each year, sometimes with consequences ranging from fractures to loss of independence.^3,4^ Most falls are multifactorial, including impaired strength, gait, and balance; use of high-risk medications; orthostatic or postural hypotension; foot or footwear issues; and visual impairments.^5^ Multifactorial interventions can help improve these risk factors, but prior pragmatic trials yielded mixed results for reducing fractures, serious injuries, and overall fall rates;^6–8^ in real-world settings, prevention strategies may be even less effective.

Prior studies have identified barriers (e.g., limited resources, competing care demands, provider knowledge, and patient motivation) and facilitators (e.g., patient engagement, financial incentives, and interprofessional collaboration) to implementing effective fall prevention clinics in primary care and consultative settings.^9–12^ However, the downstream effectiveness of these clinics, particularly in terms of follow-through for recommendations and referrals, remains understudied. Tracking real-world process measures, such as completion of referrals to physical therapy or reducing fall risk-increasing drugs (FRIDs),^13,14^ is important to understand how well interventions translate into actionable care. These measures serve as links between risk assessment and clinical outcomes, informing opportunities for potential areas for quality improvement.

The UCLA Geriatrics Fall Prevention Clinic is an interdisciplinary consultative clinic informed by evidence-based fall prevention practices,^5,6,15^ bringing together a nurse, physical therapist, geriatric medicine fellow, and attending geriatrician to conduct comprehensive fall risk assessments and deliver targeted recommendations for each patient’s modifiable risk factors. This study evaluated the effectiveness of the clinic by examining the extent to which recommendations were implemented and assessing subsequent fall- or fracture-related emergency department (ED) visits and hospitalizations.

## METHODS

### Study Design and Setting

This retrospective and pragmatic pilot study evaluated patients seen at the UCLA Geriatrics Fall Prevention Clinic between November 2020 and December 2022. The UCLA Institutional Review Board determined that this evaluation did not involve human subjects research. Patients were referred to this clinic by their primary care physician, on discharge from the hospital, or by a UCLA specialty provider. Patients were at risk of falling as determined by the referring provider, afraid of falling, or had fallen previously.

### Interdisciplinary Geriatrics Fall Prevention Clinic

Each 60-min appointment begins with screening assessments performed by the nurse, including a medication reconciliation, inquiry of self-reported falls, orthostatic blood pressure check, Fracture Risk Assessment Tool (FRAX) score calculation,^16,17^ Snellen eye chart examination,^18^ and Mini-Cog assessment.^19^ The physical therapist assessment includes assessing home safety concerns and conducting functional assessments including gait,^20^ single-leg balance,^21^ the Sit-to-Stand Test,^22^ the Forward Functional Reach Test,^23^ and ankle reactive strategy testing.^24,25^ The therapist provides recommendations for durable medical equipment, when necessary; may create a personalized home exercise program; and advises referrals for continued treatment (e.g., outpatient or home-based physical therapy).

The geriatric medicine fellow and geriatrician collect additional information from the patient and integrate nurse and physical therapist assessments to create a multifactorial care plan using a structured visit note and an electronic health record (EHR) order set. This care plan addresses polypharmacy, postural hypotension, visual impairments, feet and footwear, bone health, physical therapy recommendations, cognitive status, and social support. Educational materials, recommendations and advice, and referrals are reviewed and discussed with the patient and subsequently communicated to their primary care physician. A standardized note template and order set were developed to promote consistency in documenting evaluations and recommendations across geriatricians.

### Measures

Patient characteristics included age, gender, race and ethnicity, type of residence, living situation, whether the patient had a caregiver, and functional status using the activities of daily living (ADL) and instrumental activities of daily living (IADL) questionnaire^26^ with additional questions pertaining to mobility, transportation, and climbing a flight of stairs.

Screening measures included self-reported number of falls in the past year (positive defined as ≥ 1 fall); cognitive status (positive defined as Mini-Cog score 2 or less or known dementia diagnosis); visual acuity (positive defined as Snellen chart score 20/50 or worse in either eye, with corrective lenses if applicable); orthostatic hypotension (positive defined as a decrease in systolic blood pressure of 20 mmHg or diastolic blood pressure of 10 mmHg when standing from sitting or supine position); and fracture risk using prior bone mineral density report (positive defined as a reading of osteoporosis) or FRAX score (elevated risk for hip fracture defined as ≥ 3% or major osteoporotic fracture defined as ≥ 20%).^16,17,27^

Recommendations and orders for referrals included clinical pharmacy for further medication evaluation; ophthalmology; podiatry; osteoporosis clinic; memory clinic for more in-depth cognitive assessment or, if dementia had previously been diagnosed, the UCLA Alzheimer’s and Dementia Care program for dementia co-management;^28^ physical therapy (outpatient and home health); and occupational therapy. Documentation also included recommendations or orders to modify high-risk fall-related medications^14,29^ and to initiate bisphosphonate therapy when appropriate for osteoporosis management. Fall- and fracture-related ED visits and hospitalizations were evaluated for 1 year before (pre-) and 1 year after (post-) the fall clinic visit.

### Data Collection

Patient demographic characteristics, screening measures, and implementation of referrals and recommendations within 1 year of the clinic visit were obtained through EHR data extraction. Additional patient characteristics were abstracted from notes documented by the physical therapist and physician during the fall clinic encounter. Fall- and fracture-related ED visits and hospitalizations were identified by reviewing EHR notes, including chief complaints, ED documentation, and discharge summaries. These notes included encounters occurring within the UCLA Health system as well as outside encounters that were available through EHR interoperability tools (e.g., Care Everywhere), with the major health systems in our geographic location participating in Care Everywhere. Chart reviews were conducted by the authors (DRL, LKM, JHS, BYW, EAC, GIC, DBR) and data were entered using Research Electronic Data Capture (REDCap, Version 12.4.19).^30,31^ Chart reviewers double-abstracted 26% of charts to refine coding rules and resolved discrepancies through discussion.

### Statistical Analyses

Descriptive statistics were presented as percentages and means with standard deviations (SD). The denominators for implementation rates varied depending on the number of recommendations or referrals made during the fall clinic visit. To compare pre- and post-clinic evaluation fall- or fracture-related ED and hospitalization visits, a negative binomial regression model with clustering at the patient level was used, adjusting for patient sex and age at clinic visit. Results were presented as incidence rate ratios (IRRs) with 95% confidence intervals (CIs). All tests were two-sided with statistical significance level set at *p* < 0.05. Statistical analyses were performed using Stata/SE version 18 (StataCorp, College Station, TX 77845).

## RESULTS

### Patient Characteristics

The 94 patients included in this pragmatic pilot study were on average 79 years old; mostly women (71%); non-Hispanic White (60%); lived in a private residence (59%) with a spouse, family member, or other person (69%); and did not have a caregiver (59%) (Table 1). Regrading functional status, 39% required assistance with at least one ADL, 62% with two or more IADLs, 45% with traveling beyond walking distance, 39% with walking a quarter mile, and 29% with climbing a flight of stairs.

**Table 1:** Baseline Patient Characteristics of the UCLA Geriatrics Falls Prevention Clinic from November 2020 to December 2022

Patient characteristics	Sample (*n* = 94)
Age in years, mean (SD, range)	79.3 (8.4, 63–101)
Female sex, *n* (%)	67 (71%)
Self-reported race and ethnicity, *n* (%)	
White, not Hispanic	56 (60%)
Black or African American, not Hispanic	8 (9%)
Asian, not Hispanic	9 (10%)
Hispanic or Latinx	13 (14%)
Other^a^	8 (9%)
Type of residency, *n* (%)	
Single family house	38 (40%)
Apartment/condo	18 (19%)
Assisted living facility (ALF)	7 (8%)
Other (e.g., mobile home, hotel, triplex)	3 (3%)
Not listed	28 (30%)
Living situation, *n* (%)	
Living alone	24 (26%)
Living with spouse, family or caregiver, *n* (%)	65 (69%)
Not listed	5 (5%)
Caregiver, *n* (%)	
Spouse, children, or paid non-family	32 (34%)
None	55 (59%)
Not listed	7 (8%)
Functional status, *n* (%)	
Help with ≥ 1 ADL^b^	37 (39%)
Help with ≥ 2 IADL^c^	58 (62%)
Help with getting places beyond walking distance (e.g., taxi, bus, car), *n* (%)	42 (45%)
Help with walking ¼ mile, *n* (%)	37 (39%)
Help with climbing a flight of stairs, *n* (%)	27 (29%)

^a^Other = Not Hispanic, American Indian or Alaska Native, other race, do not identify with race, declined to answer

^b^ADL (6 items total): feeding, getting from bed to chair, getting to the toilet, getting, bathing or showering, walking across the room

^c^IADL (8 items total): using the telephone, taking medications, preparing meals, managing money, housework, shopping for personal items, shopping for groceries, driving

### Screening Assessments

**Figure 1:**
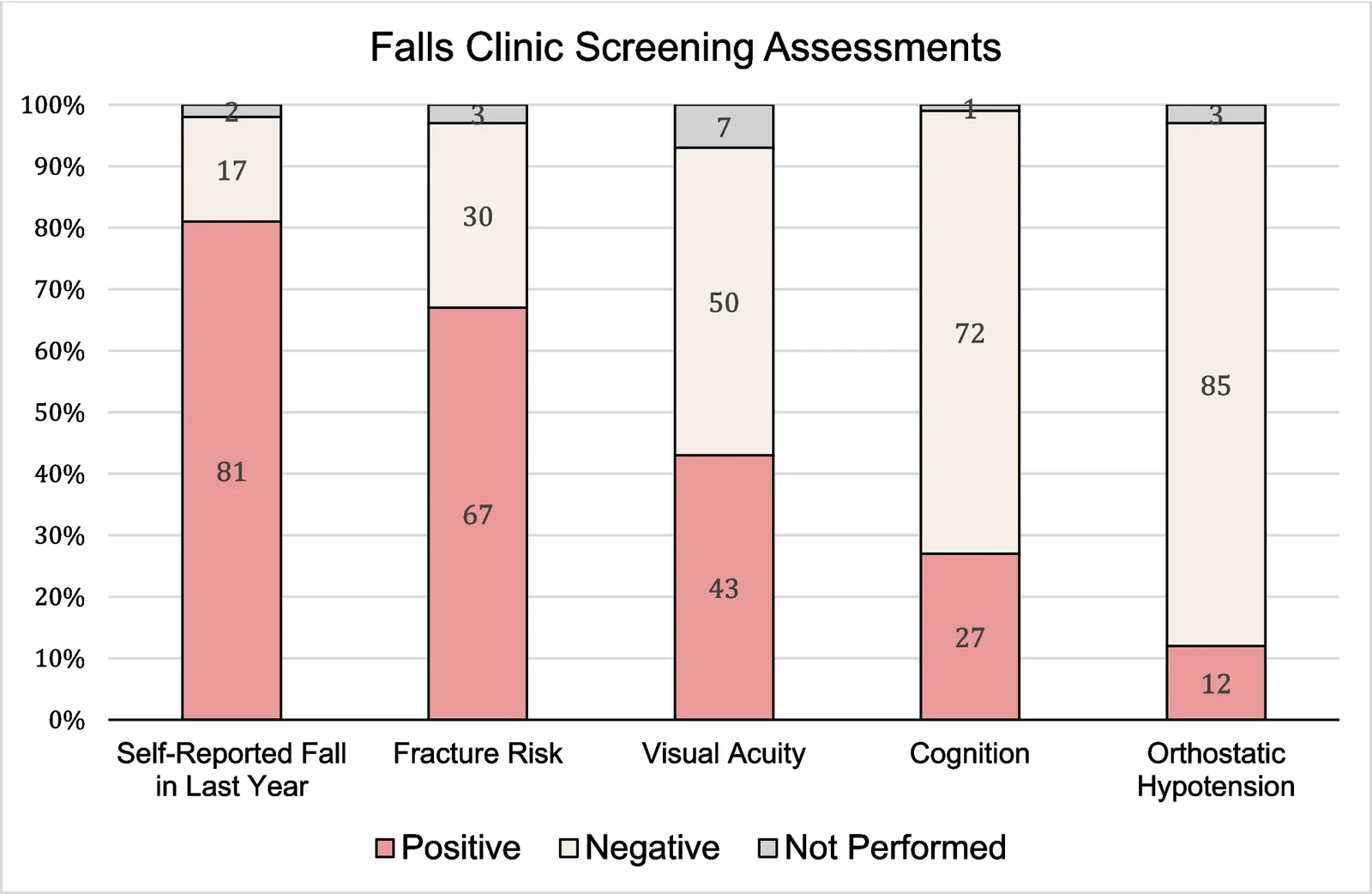
Screening assessments of falls prevention clinic participants from November 2020 to December 2022. The proportion of patients with positive (red), negative (white), and not performed or information not available (gray) results across key fall-related risk screening assessments. Percentages represent the distribution of outcomes within each screening category. Positive screens defined as: ≥ 1 self-reported fall in the last year, osteoporosis on bone density scan or elevated risk for hip fracture ≥ 3% or major osteoporotic fracture ≥ 20% using FRAX, visual acuity of 20/50 or worse, Mini-Cog score ≤ 2 or existing dementia diagnosis, decrease in systolic blood pressure of 20 mmHg or diastolic blood pressure of 10 mmHg when standing from sitting or supine position.

Assessments generated a mean 2.3 (SD = 1.1) positive screens per patient; 90/94 (96%) had at least one risk factor identified by the clinic. Most patients reported one or more falls (81%), with 64% of patients reporting two or more falls in the last year before being seen in the clinic (Fig. 1). Elevated fracture risk was identified in 67% of patients: 64% screened positive for hip fracture risk by FRAX, 48% had osteoporosis or osteopenia on DEXA scan, and 27% screened positive for major osteoporotic fracture risk by FRAX. Positive screenings were also identified for visual impairment (43%), cognitive concerns (27%), and orthostatic hypotension (12%).

### Recommendations and Subsequent Implementation Rates

**Figure 2:**
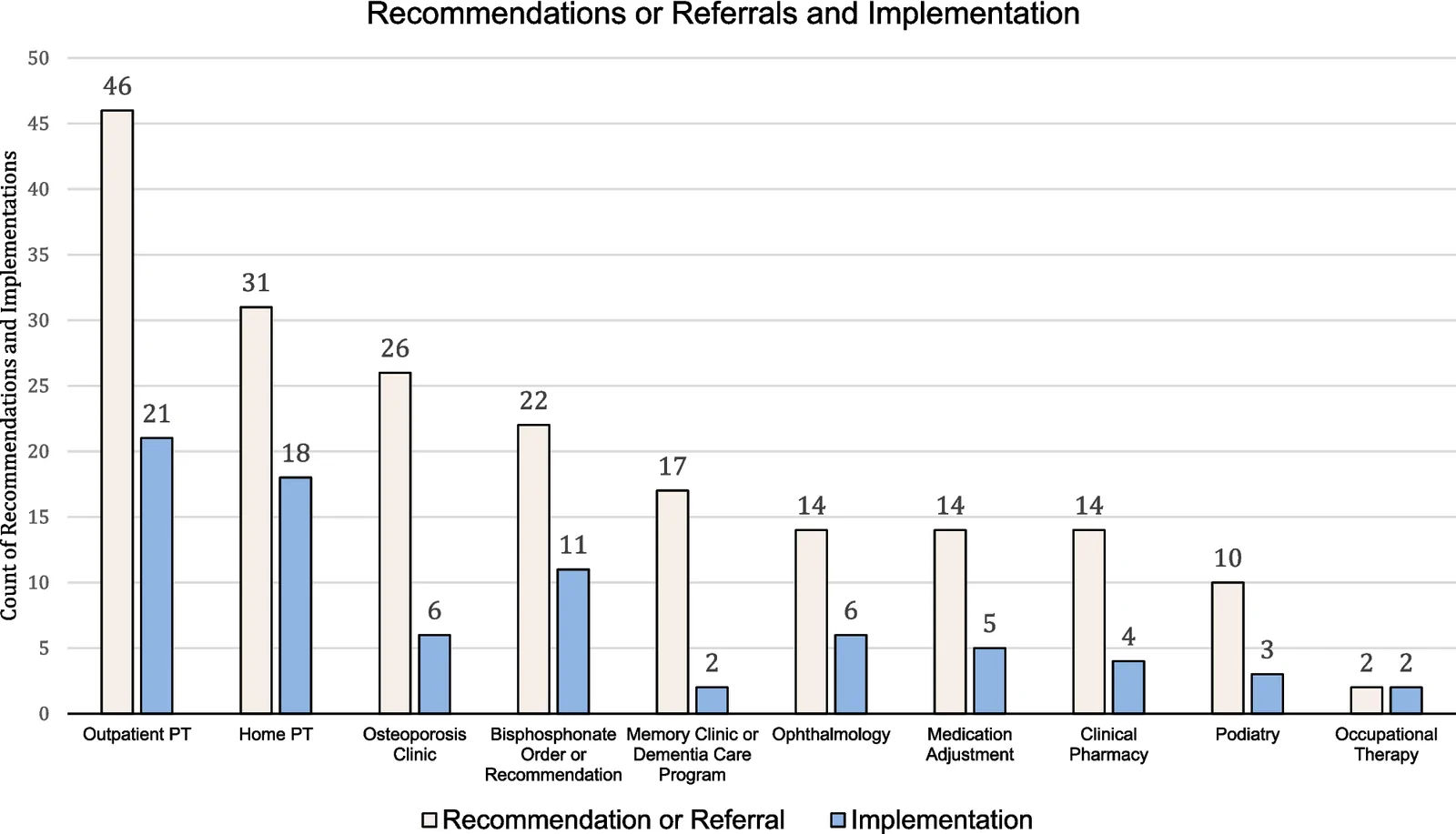
Recommendations and implementation up to 1 year after falls prevention clinic. This figure illustrates the number of patients for whom specific referrals or recommendations were made (white bars) and the number of patients who followed through with those referrals (blue bars).

A mean 2.1 (SD = 1.3) recommendations were generated per patient and 40% (78/196) were implemented. Physical therapy was the most recommended referral (Fig. 2). Among those referred, 58% (18/31) of patients who were referred to home physical therapy and 46% (21/46) of patients who were referred to outpatient physical therapy implemented the referral within 1 year of the fall clinic evaluation. Additionally, based on mobility assessments, walkers were ordered at the fall clinic visit for four patients and a cane for one patient.

Although 50% (11/22) of patients received a bisphosphonate order within 1 year of the fall clinic recommendation, only three bisphosphonate orders were placed by the fall clinic geriatrician. Implementation rates were 43% (6/14) for ophthalmology, 36% (5/14) medication adjustment recommendations, 30% (3/10) for podiatry, 29% (4/14) for clinical pharmacy, 23% (6/26) for osteoporosis clinic, and 12% (2/17) for memory clinic. Only two occupational therapy referrals were recommended and both were implemented.

### Fall-Related Healthcare Utilization Measures

After adjusting for patient sex and age at the clinic visit, there was no statistically significant difference in fall- or fracture-related ED visits in the year after the clinic compared with the year before (IRR = 0.61; 95% CI, 0.33–1.15; *p* = 0.13) (Table 2). Similarly, there was no statistically significant difference in fall- or fracture-related hospitalizations pre- and post-fall clinic visit (IRR = 0.75; 95% CI, 0.32–1.72; *p* = 0.50).

**Table 2:** Rate of Fall-Related Emergency Department Visits and Hospitalizations 1 Year Pre- vs Post-Falls Prevention Clinic

Healthcare utilization measures	Incidence rate ratio (IRR)	95% confidence interval (CI)	*p*-value
Fall- or fracture-related ED visits (ref: Pre)^a^	0.61	0.33–1.15	0.13
Fall- or fracture-related hospitalization visits (ref: Pre)^a^	0.75	0.32–1.72	0.50

^a^Negative binomial regression models adjusted for patient sex and age at clinic visit

## DISCUSSION

This evaluation demonstrated challenges of implementing an effective interdisciplinary geriatric fall prevention consultative clinic in a real-world setting. Although almost all patients had identifiable fall risk factors, especially prior falls and elevated fracture risk, fewer than half of the recommendations were implemented. Referral implementation rates for podiatry, clinical pharmacy, osteoporosis clinic, and memory clinic were even lower. Although this study did not observe statistically significant reductions in fall or fracture-related ED visits or hospitalizations, overall utilization rates were low pre- and post-clinic evaluation and the number of patients evaluated was insufficient to exclude a clinically meaningful effect.

This evaluation adds to the literature by focusing not just on risk factor identification but also on whether recommended interventions were implemented. A previous study evaluating patient engagement using secondary data from STRIDE participants^6^ found that only 33–55% of participants recalled at least one fall risk mitigation strategy.^32^ Our study complements those findings by using EHR data abstraction to objectively assess implementation on referrals and treatment recommendations, thus providing direct assessment of how fall prevention recommendations translate into receipt of recommended care. In addition, there may be larger policy-related barriers that limit follow-through on clinic recommendations. For example, fee-for-service Medicare coverage for podiatry services is restricted to diabetes-related foot care and select medical indications and does not routinely cover treatment for conditions such as painful calluses that can contribute to falls.^33^ As a result, patients may face out-of-pocket costs, which may reduce adherence to referrals despite clinically appropriate recommendations.

Because physical therapy and bone health interventions may be highest-yield strategies for fall and fracture prevention,^5,34^ increasing implementation rates in these areas is a priority. Insights from a previous fall prevention study underscore the importance of examining implementation barriers, as variability in primary care provider engagement, competing patient priorities, and clinic infrastructure were found to substantially influence uptake and effectiveness of fall prevention strategies.^12^ Possible strategies to improve implementation rates include assigning a dedicated care coordinator to assist with referral follow-up and appointment scheduling, implementing automated follow-up calls or patient portal reminders, and redesigning the clinic as a longitudinal service with in-person or virtual follow-ups to support completion of recommendations (e.g., empowering the fall clinic physician to directly implement recommendations such as deprescribing high-risk medications and starting osteoporosis treatment). Direct implementation of recommendations (e.g., ordering bisphosphonates rather than referral to osteoporosis clinic) may reduce financial and logistical burdens for patients and lead to increased adherence with recommendations. In addition, collaborating with informatics teams to embed clinical decision support tools^35^ in the EHR could help flag patients with incomplete implementation and prompt timely follow-up. These strategies could enhance the clinical impact of interdisciplinary fall prevention clinics. The ongoing tracking of process measures during clinic improvements will be important to evaluate progress and guide future quality improvement efforts.

This study had several strengths. First, it evaluated a real-world, interdisciplinary fall prevention clinic that integrates geriatrics, nursing, and physical therapy, an approach similar to other fall prevention clinics.^15^ Additionally, the use of standardized and widely adopted screening tools for fall-related risk factors supports shared learning, promoting the dissemination of successful quality improvement strategies.

This quality improvement project also had several limitations. First, as a pilot evaluation, the small sample size and low incidence of fall-related healthcare utilization limited the study’s power to detect statistically significant changes; however, future quality improvement or research efforts may include larger patient samples to better assess clinic impact. Additionally, this study was conducted at a single academic medical center and the findings may have been influenced by organization-specific factors such as referral workflows and staffing. Also, the 12-month follow-up period may have been too short to capture the full impact of fall prevention strategies, particularly for ED visits and hospitalizations. Future evaluations could consider an extended follow-up duration and incorporate statistical process control methods, such as Shewhart charts,^36^ enabling more nuanced detection of shifts in utilization patterns that may result from the intervention. In addition, although screening assessments were protocolized and most patients completed required screenings, modest variability and occasional missing assessments (1–7%) remained, which can be further investigated in future studies. Lastly, data on ED visits and hospitalizations were collected through EHR chart review; encounters at hospitals outside of UCLA, not accessible through EHR interoperability tools (e.g., Care Everywhere) may have been missed, potentially leading to an underestimation of utilization. Although most major health systems in our region participate in Care Everywhere, future studies could use Medicare claims data to more comprehensively detect fall injuries.^37^

## CONCLUSIONS

Although fall prevention clinic can identify modifiable risk factors, low implementation of key recommendations remains a barrier to the effectiveness, even at a large academic medical center. Targeted strategies to increase implementation of recommendations and ongoing quality improvement efforts can help bridge the gap between screening assessments and action, and fulfill the potential of fall prevention efforts.

## Data Availability

These data are not publicly available due to institutional restrictions and patient privacy. Deidentified data may be made available from the corresponding author upon request and permission from our institution.
